# Cryosectioning of Hydrogels as a Reliable Approach to Increase Yield and Further Tune Mechanical Properties

**DOI:** 10.3390/gels9100834

**Published:** 2023-10-20

**Authors:** África Martínez-Blanco, Sergio Noé, Lourdes Carreras-Vidal, Jorge Otero, Núria Gavara

**Affiliations:** 1 Unitat de Biofísica i Bioenginyeria, Facultat de Medicina i Ciències de la Salut, Universitat de Barcelona, 08036 Barcelona, Spain; africa.martinez@ub.edu (Á.M.-B.); sergio.noe@ub.edu (S.N.); lcarrevi29@alumnes.ub.edu (L.C.-V.); jorge.otero@ub.edu (J.O.); 2 The Institute for Bioengineering of Catalonia (IBEC), 08028 Barcelona, Spain; 3 The Barcelona Institute of Science and Technology (BIST), 08028 Barcelona, Spain; 4 CIBER de Enfermedades Respiratorias, 28029 Madrid, Spain

**Keywords:** hydrogel, genipin, decellularization, extracellular matrix, cryosectioning, cryogel, Young’s modulus, atomic force microscopy, stiffness, sterilization

## Abstract

Decellularized extracellular matrix (dECM) hydrogels have emerged as promising materials in tissue engineering. The steps to produce dECM hydrogels containing the bioactive epitopes found in the native matrix are often laborious, including the initial harvesting and decellularization of the animal organ. Furthermore, resulting hydrogels often exhibit weak mechanical properties that require the use of additional crosslinkers such as genipin to truly simulate the mechanical properties of the desired study tissue. In this work, we have developed a protocol to readily obtain tens of thin dECM hydrogel cryosections attached to a glass slide as support, to serve as scaffolds for two-dimensional (2D) or three-dimensional (3D) cell culture. Following extensive atomic force microscopy (AFM)-based mechanical characterization of dECM hydrogels crosslinked with increasing genipin concentrations (5 mM, 10 mM, and 20 mM), we provide detailed protocol recommendations for achieving dECM hydrogels of any biologically relevant stiffness. Given that our protocol requires hydrogel freezing, we also confirm that the approach taken can be further used to increase the mechanical properties of the scaffold in a controlled manner exhibiting twice the stiffness in highly crosslinked arrays. Finally, we explored the effect of ethanol-based short- and long-term sterilization on dECM hydrogels, showing that in some situations it may give rise to significant changes in hydrogel mechanical properties that need to be taken into account in experimental design. The hydrogel cryosections produced were shown to be biocompatible and support cell attachment and spreading for at least 72 h in culture. In brief, our proposed method may provide several advantages for tissue engineering: (1) easy availability and reduction in preparation time, (2) increase in the total hydrogel volume eventually used for experiments being able to obtain 15–22 slides from a 250 µL hydrogel) with a (3) reduction in scaffold variability (only a 17.5 ± 9.5% intraslide variability provided by the method), and (4) compatibility with live-cell imaging techniques or further cell characterization of cells.

## 1. Introduction

Tissue engineering and the accurate recreation of cellular function in vivo involves the development of culture scaffolds capable of mimicking the physiological and pathological extracellular matrix (ECM). The engineered ECM should not only provide structural support, but also serve as a source of physicochemical and biomechanical stimuli specific to the cells under study [1,2]. Among the scaffolds developed for tissue engineering, hydrogels stand out: 3D crosslinked polymer networks characterized by their hydrophilic properties [3,4,5,6], high porosity, and hydration (>30% water by weight) producing structures with a degree of elasticity similar to the ECM of living tissue [5,7]. Much of the research on hydrogels has been based on those of synthetic origin (such as polyethylene oxide, polyethylene glycol, or polyacrylamide); however, these are limited in the recreation of many fundamental biological functions and signaling [6,8]. A new generation of decellularized ECM (dECM) hydrogels of the target tissue as in [9,10] are being developed, but further understanding is needed on the synthesis, crosslinking, and preservation of hydrogels over time or the effects of sterilization to generate dECM scaffolds that closely simulate the cellular microenvironment.

To produce dECM hydrogels, the ECM is decellularized, freeze-dried, milled, and processed into a digested and neutralized powder, to obtain a thermosensitive pregel that is polymerizable at 37 °C [11]. While the biochemical properties of the ECM are mostly maintained with such hydrogels, it remains a challenge to mimic the mechanical properties after gelation [12], likely due to the different organization of the dECM hydrogel in thinner fibers as compared to native tissue [13]. Accordingly, several crosslinking agents have been proposed [13,14]—among them, genipin is in the spotlight. This geniposide aglycone from gardenia fruit (*Gardenia jasminoides* Ellis) has low toxicity [12,15,16], immunogenicity in vivo [17], and high degree of crosslinking [14]. Although increased biomechanical efficacy has been demonstrated, concentrations and crosslinking times required to recreate mechanical features of any given tissue remain unknown.

Existing protocols for the generation of dECM hydrogels from powder are often cumbersome, requiring long digestion, synthesis, and crosslinking times. As such, the pregel is typically stored at −80 °C until gelation and crosslinking are carried out. When these steps are performed, the output is often a large macroscopic gel, even when only a thin microscopic-scale gel surface is required or even preferred for cell culture and imaging. In our laboratory, we have developed optimized protocols for the cryosectioning of whole animal organs samples, using them for further cell culture, imaging, or mechanical characterization [18,19,20]. One of the key advantages is the ability to expand the number of samples that can be used from scarce tissue [18]. Accordingly, we assessed whether the cryosectioning approach could be used in our crosslinked dECM hydrogels, in order to reduce processing time, increase the volume of sample eventually used, reduce sample variability, and allowing us to apply different cell and dECM characterization techniques on the same sample (e.g., cell culture, mechanical characterization, and immunostaining, among others). Here, we present a method for cryosectioning crosslinked hydrogels attached to glass slides that maintain the same properties as a fresh gel, allowing subsequent cell culture and different molecular biology and biophysical techniques. Due to the extensive experience of our laboratory in lung tissue, we chose to focus on lung dECM hydrogels (L-dECM), crosslinked with genipin, with the aim of characterizing the gel/crosslinker concentrations and their resulting mechanical properties. We have further characterized the effects of hydrogel long-term storage and sterilization, to propose a comprehensive protocol for dECM hydrogels synthesis, storage, and refinement for tissue engineering.

## 2. Results and Discussion

### 2.1. Characterization of Genipin Crosslinked L-dECM Hydrogels

**Figure 1:**
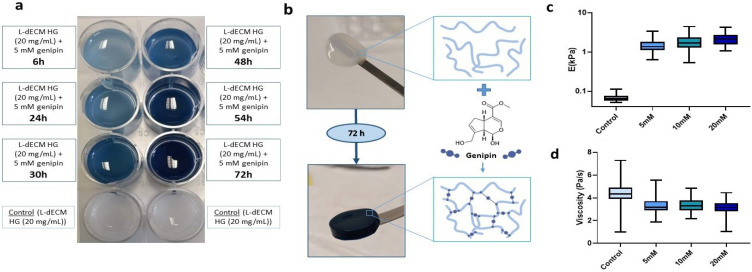
Effect of genipin crosslinking on L-dECM hydrogels. (**a**) Characteristic bluish staining of genipin (20 mg/mL + 5 mM genipin) time-dependent crosslinking compared to control (20 mg/mL); (**b**) Macroscopic images of the structural integrity of the control hydrogel (20 mg/mL) and hydrogel crosslinked with genipin (20 mg/mL + 5 mM genipin) and schematic illustration of the genipin cross-linking reaction; (**c**) Results of Young’s modulus (E) and (**d**) viscosity depending on the concentration of genipin administered to the hydrogels.

To determine the effect of genipin crosslinking on the mechanical stability of our lung hydrogels, a panel of L-dECM hydrogels (20 mg/mL) was prepared and crosslinked for 72 h in a genipin solution at different concentrations (5 mM, 10 mM, and 20 mM). The degree of crosslinking was visible by naked eye as shown by the acquisition of a bluish hue that increased to a maximum after 72 h (Figure 1a). This was accompanied by an increase in the structural integrity of the hydrogels, allowing their complete and easy manual manipulation, regardless of the concentration of crosslinker solution (Figure 1b).

We performed mechanical characterization of the hydrogels by carrying out force-indentation curves using AFM. We measured hydrogel stiffness (reported throughout the manuscript as Young’s modulus (E)) and viscosity at 25 °C in 1X phosphate buffered saline (PBS). Non-crosslinked hydrogels were measured on the same day of gelation while crosslinked hydrogels were measured 72 h after addition of genipin. As expected, we observed a 21-fold increase at 5 mM (1.36 kPa (1.11–1.82 kPa)), 26.3-fold increase at 10 mM (1.70 kPa (1.30–2.35 kPa)), and 32.8-fold increase at 20 mM (2.12 kPa (1.56–2.70 kPa)) in stiffness with respect to the uncrosslinked hydrogels (0.065 kPa (0.058–0.76 kPa) (Figure 1c).

### 2.2. Diffusion Pattern of Genipin into the Hydrogel

**Figure 2:**
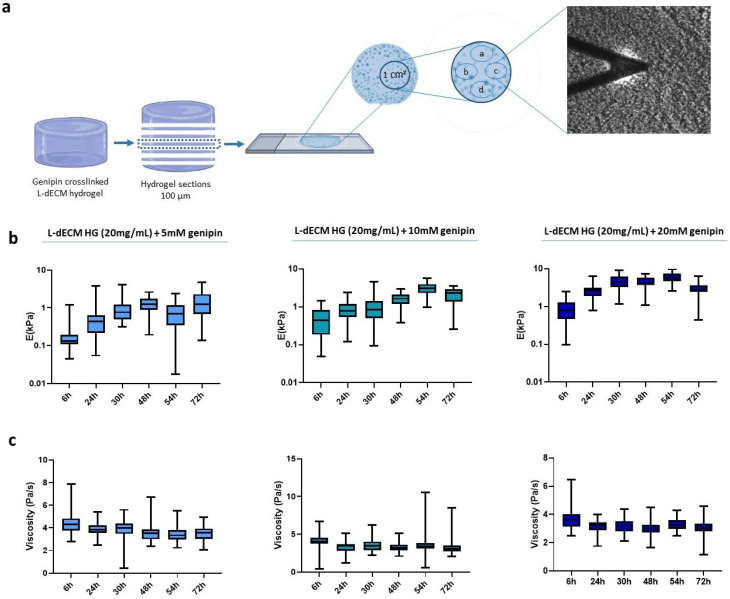
Diffusion pattern of genipin inside the hydrogel. (**a**) Representative image of the experimental methodology followed to determine the diffusion study areas; (**b**) Stiffness of the hydrogel slides; (**c**) Viscoelasticity depending on the crosslinking time obtained by AFM force-indentation curves.

We next studied the diffusion of genipin into the macroscopic hydrogel by creating a panel of L-dECM hydrogels that were crosslinked with genipin solutions (5 mM, 10 mM, and 20 mM) but for which the process was stopped after different time periods. Then, we froze the hydrogels according to the freezing protocol and stored them at −80 °C. To reach the centermost region of the hydrogel, that is, the area where genipin would take longer to diffuse into, we carried out the following procedure. Knowing the volume and diameter of the well, we calculated the expected height of the hydrogel and sliced it multiple times until we reached the central region of the hydrogel (in z). On that hydrogel slice, we established a study area of 1 cm^2^ in the central region (in x-y) of each section and measured it by AFM in four nearby regions (15–20 points/sample), using a spatial distribution that was maintained throughout the experiment (Figure 2a).

We find that the stiffness of the crosslinked hydrogels increased in all cases with respect to crosslinking time, displaying a peak at 48 h for the 5 mM solution and at 54 h for 10 mM and 20 mM (Figure 2b). However, at 72 h there is an unexpected decrease in the Young’s modulus of hydrogels crosslinked with 10 mM and 20 mM. In addition, we can observe a linear decreasing trend of the viscoelasticity of 17.2% for 5 mM, 23.6% for 10 mM, and 16.8% for 20 mM (Figure 2c).

### 2.3. Effect of Cryosectioning on the Mechanical Stability of Genipin-Crosslinked dECM Hydrogels

**Figure 3:**
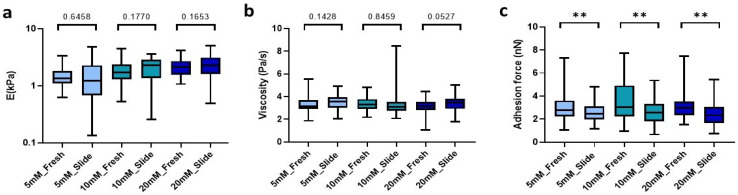
Comparison between the hydrogel crosslinked with the different genipin solutions under study and the hydrogel cryosections developed. (**a**) E; (**b**) Viscoelasticity and (**c**) Adhesion force of the slides. Significance is shown as ** *p*-value < 0.01 and non-significance is shown by the *p*-value.

The method presented here requires freezing of our macroscopic hydrogels in order to easily slice them into microns-thin slices. Accordingly, we verified whether the process of optimum cut-off temperature compound (OTC) inclusion, freezing, and subsequent rehydration affected the mechanical properties of our hydrogels. Using a cryostat, we obtained 100 µm sections that were rehydrated with 1X PBS and subsequently measured by AFM at 25 °C. Of note, we found no significant differences in either stiffness and viscosity (Figure 3a,b) between the hydrogel cryosections and their fresh counterparts. Nevertheless, we did observe significant differences in adhesion force, which decreased by 11% (*p* = 0.0082) in low-concentration crosslinked slides (5 mM), while in denser matrix slides (10 mM and 20 mM) the adhesion force was reduced by up to 16.2% (*p* = 0.0037) and 22.4% (*p* = 0.0019), respectively, compared to fresh hydrogels with the same crosslinking conditions (Figure 3c).

### 2.4. Analysis of the Critical Point of Slide Rehydration

**Figure 4:**
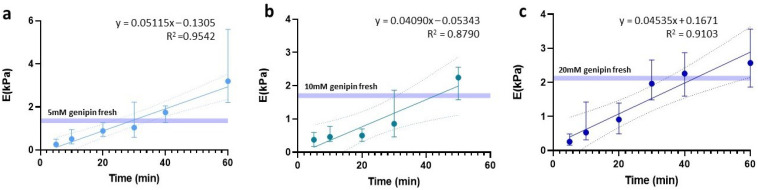
Linear regression of stiffness dependent on time prior to sample rehydration. Regressions are expressed for (**a**) 5 mM, (**b**) 10 mM, and (**c**) 20 mM.

One of the critical points for hydrogel slides to maintain a stiffness value similar to their fresh counterparts lies in finding an optimal balance between OCT removal and slide rehydration, to avoid it from drying out. To this end, we assessed the effect of rehydration time with 1X PBS for the three hydrogel concentrations under study (5 mM, 10 mM, and 20 mM). Since we had no specific hypothesis about the temporal behavior of the data, linear, logarithmic, and exponential trends were tested and based on the highest R^2^ obtained for all three, a linear fit was chosen (Figure 4).

Using this fit, we determined the waiting time required to rehydrate the slides in order to maintain the same stiffness as the fresh hydrogels. In the case of 5 mM, the rehydration was 29.1 min, reaching a significantly larger stiffness value than the fresh counterpart (*p* = 0.0002) if we waited up to 40 min. Meanwhile, for the 10 mM and 20 mM slides, we established an optimal rehydration time of 43 min.

### 2.5. Sources of Variability of dECM Hydrogel Cryosections Crosslinked with Genipin

The method presented here aims at obtaining a large number of slides of the same hydrogel, thus increasing sample output while reducing sample variability in our experiments. In order to assess which are the dominant sources of stiffness variability affecting our slides, we obtained samples of two hydrogels crosslinked with a 5 mM genipin solution for 72 h prepared on different days. All samples were measured by AFM using the same distribution of measurement zones, and we computed the covariance for each of the sources of variability studied. According to our results, the largest source of variability came from local mechanical heterogeneity in the sample itself (intraslide), followed by the processing of the crosslinked hydrogels by freezing, cutting, and rehydration (interslide). Finally, the smallest sources of variability were given by the production of our bulk hydrogels (intergel) as well as from the force-indentation probing and data acquisition with the AFM (intrarramp) (Table 1).

### 2.6. Effect of Freezing on the Mechanical Stability of Genipin-Crosslinked dECM Hydrogels

**Figure 5:**
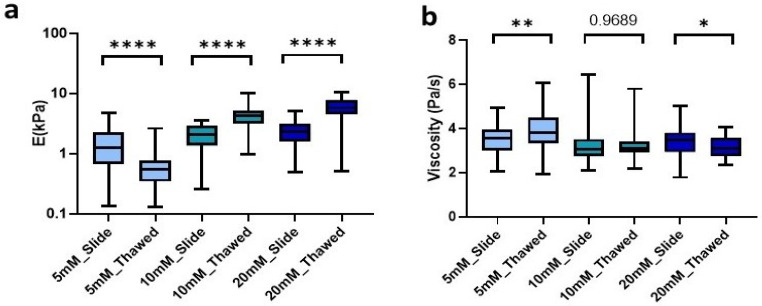
Effect of freezing on (**a**) stiffness and (**b**) viscoelasticity of cryosections of L-dECM hydrogel crosslinked with genipin. Significance is shown as * *p*-value < 0.05, ** *p*-value < 0.01, **** *p*-value < 0.0001 and non-significance with the *p*-value.

To further explore the ease and applicability of the hydrogel cryosections developed in this manuscript, we set out to analyze the effect of freezing on them. To this end, we produced 100 µm hydrogel slides that were frozen at −80 °C for at least 24 h. Subsequently, they were allowed to thaw at room temperature (RT) for the time determined above for each of our conditions and then analyzed mechanically. We observed that freezing lead to significant changes of the Young’s modulus in all conditions. However, the changes were different depending on the degree of crosslinking. Matrices crosslinked with 5 mM genipin solution experienced a significant (*p* < 0.0001) decrease in stiffness of 55.6% from 1.24 kPa (0.68–2.28 kPa) in unfrozen sections to 0.55 kPa (0.34–0.8 kPa) in frozen sections. Likewise, by increasing the concentration of genipin, we could observe this time a significant increase (*p* < 0.0001) in stiffness by 105.2% for 10 mM going from 2.06 kPa (1.35–2.88 kPa) to 4.23 kPa (3.18–5.13 kPa) and 154.5% for 20 mM, showing a median of 2.28 kPa (1.61–3.12 kPa) in unfrozen sections to 5.81 kPa (4.52–7.82 kPa) in frozen ones. As observed in unfrozen samples, the concentration of crosslinker showed a positive correlation with resulting stiffness in the probed cryosectioned samples (Figure 5a).

Finally, we were also able to observe similar changes in the viscosity of the matrices due to freezing. On the one hand, the 5 mM crosslinked matrices experienced a significant increase (*p* = 0.0058) in viscosity of 0.25 Pa/s in an inverse relationship to the effect of freezing on stiffness. On the other hand, matrices crosslinked with 20 mM genipin experienced a decrease (*p* = 0.037) in viscosity of 0.37 Pa/s. However, the 10 mM cross-linker was the only one that did not experience significant changes due to the effect of freezing and thawing of the sample (Figure 5b).

### 2.7. Effect of Sterilization Treatments on Genipin-Crosslinking dECM Hydrogels

**Figure 6:**
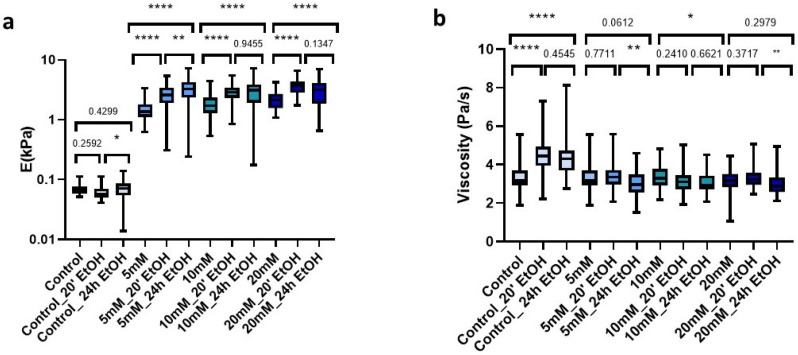

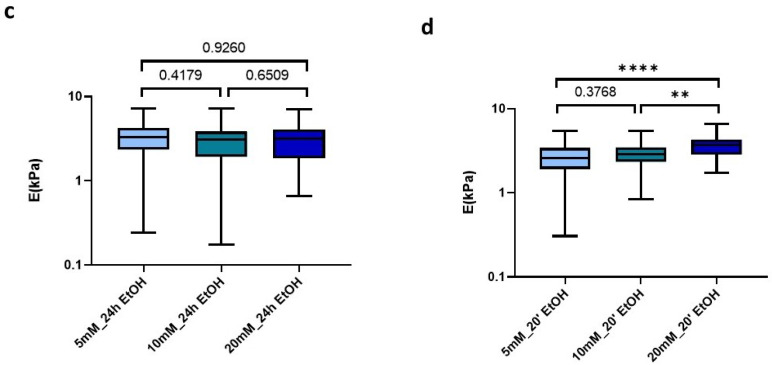
Effect of sterilization time with EtOH 70% on (**a**) E; (**b**) Viscosity in control and crosslinked L-dECM hydrogel with genipin; (**c**) Loss of identity with 24 h sterilization time and (**d**) with 20 min; (**d**) Loss of identity with 24 h sterilization time. Significance is shown as * *p*-value < 0.05, ** *p*-value < 0.01, **** *p*-value < 0.0001 and non-significance with the *p*-value.

The improved mechanical properties due to genipin made our dECM hydrogels suitable candidates for use as cell culture scaffolds. However, the required use of sterilant on the matrix prior to cell culture may induce changes in the mechanical rigidity. Accordingly, we studied the changes in stiffness and viscosity of hydrogels both with control and genipin-crosslinker under short and long time periods (20 min and 24 h) with a 70% EtOH solution. For this, we first measured the hydrogels prior to AFM sterilization. Then, we immersed the hydrogels in the sterilization solution (EtOH 70%) and subsequently performed three washes with 1X PBS and proceeded to measure them again. The uncrosslinked matrices did not experience significant changes with any of the sterilization times (Figure 6a); however, they did experience significant changes in viscosity (*p* < 0.0001) with both treatments (Figure 6b). In contrast, all hydrogels to which we added crosslinker experienced significant changes (*p* < 0.001) in stiffness, which was dependent on sterilization time. For genipin-crosslinked hydrogels, they experienced a gradual time-dependent increase in stiffness as compared to their fresh counterparts (Figure 6a). As for the viscosity of the crosslinked matrices, we could observe that treatment with 70% EtOH for 20 min did not produce significant changes in any of them (5 mM (*p* = 0.771), 10 mM (*p* = 0.241), and 20 mM (*p* = 0.371)). In addition, for longer sterilization periods (24 h), viscosity was only significantly affected (*p* = 0.0419) in the 10 mM matrices, while in the rest we could observe that there were no significant changes compared to non-sterilized crosslinked hydrogels (Figure 6b).

Analyzing in more detail the applied treatments, we could observe that the use of 70% EtOH for 24 h induced all matrices to display very similar stiffness values, despite the amount of genipin crosslinking concentration initially used (5 mM: 3.28 kPa (2.35–4.24 kPa), 10 mM: 3.11 kPa (1.93–3.85 kPa), 20 mM: 3.14 kPa (1.87–4.09 kPa)) (Figure 6c). In contrast, this effect was less noticeable when we applied much shorter sterilization periods (20 min), which still yielded a positive correlation of stiffness with genipin concentration, as observed for the non-EtOH treated hydrogels (Figure 6d).

### 2.8. Evaluation of the Mechanical Stability of Genipin-Crosslinked dECM Hydrogels after 21 Days Post-Sterilization

Having assessed the effects of sterilization on crosslinked and non-crosslinked matrices, we set out to measure the long-term mechanical stability of sterilized matrices with the two time periods studied (20 min and 24 h). Hydrogels were sterilized as described above and then in an incubator at 37 °C and 5% CO_2_ for 21 days, with PBS being replenished every 2 days. It is worth pointing out that sterile conditions were maintained inside the incubator and during PBS exchange, and no antibiotics were used in the buffer solution throughout. After 21 days, the hydrogels were mechanically characterized, having first confirmed that no hydrogel displayed visual contamination for either sterilization treatment initially used.

**Figure 7:**
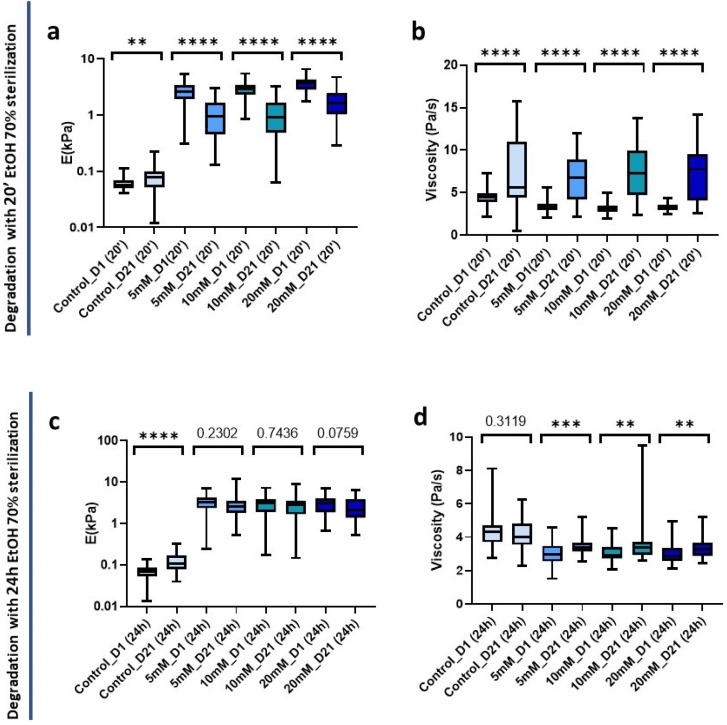
Hydrogel degradation pattern 21 days post-sterilization with 70 % EtOH. (**a**) Stiffness and (**b**) viscoelasticity of hydrogels treated with 20 min sterilization; (**c**) Stiffness and (**d**) viscoelasticity of hydrogels treated with 24 h sterilization. Significance is shown as ** *p*-value < 0.01, *** *p*-value < 0.001, **** *p*-value < 0.0001 and non-significance with the *p*-value.

Non-crosslinked hydrogels experienced minor changes and interestingly were the only ones to experience a significant increase (*p* = 0.0068) in stiffness from 0.058 kPa (0.050–0.70 kPa) to 0.077 kPa (0.052–0.1 kPa) at 21 days post-20 min sterilization. Conversely, all crosslinked hydrogels underwent a significant decrease in stiffness (*p* < 0.0001) (Figure 7a). In parallel, in all cases there was also a highly significant (*p* < 0.0001) increase in viscosity. In addition, the interquartile range increased indicating a greater dispersion of the data after 21 days of sterilization application (Figure 7b).

We also analyzed the changes in the mechanical properties of the hydrogel after 21 days post-24 h sterilization. We could observe a significant stiffness increase (*p* < 0.0001) in the control hydrogels that did not affect the viscosity (*p* = 0.3119) (Figure 7c,d). However, interestingly, we could observe that the crosslinked matrices did not suffer significant changes in stiffness at 21 days (Figure 7c), suggesting a higher stability over time than when we applied a short sterilization time.

### 2.9. Cell Behavior on the Hydrogel Slides

**Figure 8:**
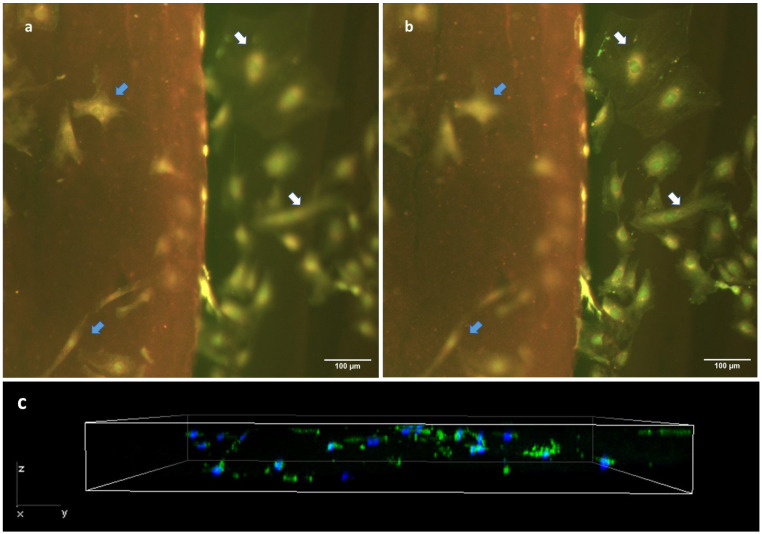
Cell behavior in cryosections of hydrogel crosslinked with 5 mM genipin. Epifluorescence images (**a**,**b**) show the same hydrogel location, but are focused on different planes. Cells have been stained with a Live/Dead kit. Image (**a**) is focused on the hydrogel top surface and shows only in focus cells that are adhered to the top of the hydrogel (blue arrows), while (**b**) is focused on the glass substrate and shows only in focus the cells that are adhered to the glass substrate (white arrows). All cells can be seen in both images (as indicated by arrows), while not simultaneously in focus. (**c**) Z-stack of cryosection with F-actin (green) and DAPI (nucleus).

Finally, we confirmed the cell-scaffolding properties of our hydrogels slides by seeding fibroblasts on them at a density of 3000 cells/cm^2^ and keeping them in culture for 72 h. We then performed a Live/Dead test to check cell viability, the proportion of cells adhered to the hydrogel compared to glass and cell morphology (Figure 8a,b). Of note, the genipin-induced fluorescence caused the matrix to be stained in red, which allowed us to clearly differentiate the substrate to which the cells were attached. We observe a high cell viability on our hydrogel cryosections with genipin. Cells were randomly distributed all over the surface of the cryosection, and no gradients of cell number or empty zones were observed in the central regions of the cryosection’s surface (blue arrows in Figure 8a). On the other hand, we could also observe a similar distribution of cells on the glass surface in the vicinity of the hydrogel (white arrows in Figure 8b). Regarding cell morphology, the cells found on the hydrogel exhibited a less stellate morphology, appearing more spherical or spindle-shaped (Figure 8a), compared to the cells deposited on the stiffer glass substrate (Figure 8b). We were also able to observe that after 72 h of culture, cells migrated into the gel, thus showing that our hydrogel cryosections can act also as 3D scaffolds (Figure 8c). In this 3D setting, cells displayed a diffuse F-actin pattern, with a morphology that, as expected, differed from that found in 2D conditions.

### 2.10. Discussion

In this work, we present a characterization of the mechanical properties L-dECM hydrogels crosslinked with different genipin solutions (5 mM, 10 mM, 20 mM). We show that genipin increases the stiffness of L-dECM proportionally to the concentration of genipin and the crosslinking time. However, other factors such as sterilization or freezing allow us to further modulate the final stiffness of the sample.

The non-crosslinked L-dECM hydrogels exhibited weak mechanical properties after gelation, which were in agreement with studies by Link et al. [12]. This may be due to three main reasons: impoverished or damaged profile of the matrix proteins after decellularization, unspecificity of the enzymatic digestion with pepsin, and/or differential fibrillar organization due to the reassembly process during gelation [12,13]. In line with studies by others [17,21,22], the use of genipin as a crosslinker allowed the improvement of the stiffness of the hydrogels while decreasing the viscosity, showing a positive correlation between the genipin content and the stiffness of the hydrogels where the crosslinker reacts with the free amino groups present in the ECM forming a highly crosslinked network. However, the fold increase in Young’s modulus observed for other types of hydrogels when crosslinked with genipin indicates that our matrix does not allow such intensive crosslinking as other materials such gelatin [23] or chitosan [22]. We have verified that in our protocol, the diffusion of genipin into the hydrogel is sufficient to guarantee that the crosslinking occurs even in the center of the hydrogel, giving rise to consistent mechanical properties throughout the depth and width of the gels. Thus, we were able to observe a crosslinking peak at 48 h for hydrogels crosslinked with 5 mM and 54 h for those crosslinked with 10 mM and 20 mM, after which a decrease in stiffness was observed. This could be consistent with the formation of porous structures inside the bioscaffolds after modification with genipin that has been previously described by several authors [24,25,26] and that after cutting could be brought to the slide surface.

One of the main disadvantages of genipin crosslinking is the generation of bluish residues during the preparation of hydrogels, limiting their transparency and their use as 3D culture systems [27]. Indeed, this crosslinker exhibits autofluorescence at 630 nm depending on the concentration of genipin [28], which can make it difficult to apply these hydrogels to, for example, confocal microscopy, epifluorescence, or immunohistochemistry. Furthermore, working with thick macroscopic samples may make the mechanical characterization of hydrogels via AFM complicated due to the difficulty to observe the cantilever by optical means through the depth of the gel. In order to address these problems, in this article we developed a method of cryosectioning genipin-crosslinked hydrogel slides to obtain in a simple way a thin and easy-to-handle sample glued to a glass slide, which, under a carefully designed protocol, maintains the same mechanical properties as fresh hydrogels. The main advantages offered by this method are as follows: (1) Ease and reduction in sample preparation time, since we only need to make the cuts with a cryostat and rehydrate the slides at the moment of hydrogel use. (2) Increase in the useful sample volume: in our case, we have been able to obtain 15–22 slides from the same hydrogel. (3) Reduction in sample variability, since it is possible to have multiple repeats from the same hydrogel. (4) Applicability of multiple techniques (e.g., cell culture, AFM, microscopy). We anticipate that thinner hydrogel slides can also be easily produced, but this would require the generation of new rehydration curves to estimate new optimized rehydration times, in order to achieve the desired mechanical properties for the final cryosectioned slices.

Freezing has been described as a physical crosslinking method capable of increasing the mechanical properties of hydrogels based on the crystallization process taking place in the hydrogel [29]. However, it should be noted that the reinforcement of the mechanical properties will depend on both the initial characteristics of the hydrogel and the characteristics of the freezing process (time, temperature, number of freeze-thaw cycles) [29,30]. Initially, we used freezing to facilitate the process of cutting our samples and thus evaluated the effect the process had on their mechanical properties. However, we found that freeze-thawing acted as a second crosslinking stage of physical origin in matrices treated with higher concentrations of genipin (10 mM and 20 mM). Previous studies on synthetic polymer-derived hydrogels (mostly polyvinyl alcohol (PVA)) have also show reinforcement of their mechanical properties after the application of freeze-thaw cycles [29,30,31]. However, in our case the increase in stiffness is not so large as that reported by others, probably because the initial macroscopic hydrogel that gives rise to the cryosections was initially pre-treated with cryoprotectant solutions to prevent the creation of ice crystals inside it. Nevertheless, our results confirm that the freeze-thaw cycle can be used for further controlled modulation of stiffness for previously cross-linked hydrogel matrices.

Soft hydrogels have been used for a plethora of purposes, including cell differentiation [23], cell culture proliferation [32], cell adhesion [33], or assessment of cell-matrix interactions [34], among others. Accordingly, sterility is required for the success of all these objectives. While it may be anticipated that the sterilization process will affect the mechanical stability of soft hydrogels, quantitative confirmation is lacking. In our tests, we used as sterilant EtOH 70%, because it is a practical, effective method and after sufficient rinsing washes less than 1% of the EtOH is thought to be retained in the hydrogel [35]. We observed that sterilization for 24 h gave rise to a saturation in the stiffness of our hydrogels, and thus prevented our ability to modulate their final stiffness by using different concentrations of genipin crosslinker. In contrast, hydrogels treated for shorter sterilization times (20 min) retained mechanical properties that correlated with genipin concentration. On the other hand, we observed that matrices sterilized for short periods of time displayed a decrease in their stiffness when kept in culture for weeks, while stiffness was maintained for matrices that had been treated with EtOH for 24 h. Together, this presents itself as a dual role played by EtOH 70% treatment, simultaneously sterilizing and preserving long-term mechanical properties. Accordingly, we recommend that the expected duration of cell culture on the hydrogels is first considered, to thus adapt the sterilization processes. In particular, when cells are expected to grow on hydrogels for short periods of time, a short sterilization process is recommended. On the contrary, when cells will be grown on hydrogels for several weeks, we recommend longer EtOH sterilization periods, which will additionally stabilize the hydrogel and prevent long-term degradation of the dECM hydrogel samples.

## 3. Conclusions

In conclusion, in this article we present for the first time a mechanical characterization of lung dECM hydrogels cross-linked with different concentrations of genipin. In addition, we develop a hydrogel cryosectioning protocol, which serves as a useful tool not only to save scaffold production time and increase the usable sample volume, but also addresses some of the challenges presented by the intrinsically weak mechanical properties of the lung dECM hydrogel. Finally, our results on hydrogel sterilization help put into context how this trivial step affects the short- and long-term mechanical stability of hydrogel samples.

## 4. Materials and Methods

### 4.1. Cell Culture

RLF-6 cells (ATCC, Manassas, VA, USA) lines were grown as per ATCC recommended culture conditions, using specific F-12 K medium (Fisher Scientifc, Florence, KY, USA) supplemented with 20% FBS (ThermoFisher Scientific, Waltham, MA, USA) and 1% penicillin-streptomycin (ThermoFisher Scientific, Waltham, MA, USA). Cells were seeded at a concentration of 3000 cells/cm^2^. Culture media was removed and replenished every 3 days prior to washing with 1X phosphate buffer saline (PBS).

### 4.2. Preparation of Lung Extracellular Matrix (L-dECM) Hydrogels

#### 4.2.1. Lung Decellularization

Porcine lungs (*Sus scrofa domesticus*) were obtained from four different animals from a local slaughterhouse. Prior to the decellularization protocol, lungs were examined to discard any damage and the connective tissues surrounding the trachea and throat were removed to expose the vascular and airway structures necessary for perfusion. The right lung was removed and a clamp was placed blocking the right main bronchus as well as the vasculature of the right airways, thus creating a closed system to facilitate perfusion of the left lung during decellularization [13]. The decellularization protocol applied has been previously described by [13] and briefly modified by [36]. Lungs were perfused with miliQ water through the trachea and vasculature. Solutions were removed passively, driven by the elasticity of the tissue by massaging gently all parts of the tissue. Triton X-100 0.1% (Sigma-Aldrich, St. Louis, MO, USA) was perfused through both routes and clamps were placed after injection to prevent clearance. The lung was placed in a container with the same solution and incubated for 24 h at 4 °C. The tissue was then perfused again using 2% sodium deoxycholate (SDC) (Sigma-Aldrich, St. Louis, MO, USA) and immersed in the same way for 24 h at 4 °C. On the last day of the protocol, a 1 M NaCl (Sigma-Aldrich, St. Louis, MO, USA) solution was perfused and incubated for 1 h at 4 °C and then the tissue was perfused again with a DNAse solution and incubated for 1 h at 4 °C. Three miliQ water washes were performed between consecutive reagent perfusions to remove solutions and cellular waste. Finally, three washes of 1X PBS were performed. All solutions were kept at 4 °C overnight prior to decellularization except for DNAse which was prepared prior to perfusion and at RT (22 °C). The perfused volumes were maintained throughout the protocol and were 1.5 L per airway and 1 L per pulmonary vasculature. Finally, the decellularized tissue was sectioned, eliminating perceptible cartilaginous areas (bronchioles), and stored at −80 °C (Telstar Lyoquest-55 Plus, Terrassa, Spain).

#### 4.2.2. Preparation of L-dECM Hydrogels

The decellularized lung fragments were freeze-dried and pulverized into micrometric particles using a cryogenic mill (6755, SPEX, Metuchen, NJ, USA) (−180 °C) for 5 min at maximum speed. An equal proportion of each lung was added to each of the pulverizations to ensure representation and homogeneous mixing of four lungs. The powder obtained was resuspended at 20 mg/mL in 0.01 M HCl (Sigma-Aldrich, St. Louis, MO, USA) (pH = 2) and digested with pepsin (1:10) (Sigma-Aldrich, St. Louis, MO, USA) under magnetic stirring (400 rpm) at RT for 16 h. Subsequently, the digestion was diluted with 10X PBS (1:9) and neutralized (7.4 ± 0.4) with NaOH (Sigma-Aldrich, St. Louis, MO, USA). All solutions used were stored at 4 °C for at least 24 h prior to neutralization and the work was carried out on ice to avoid early gelation. Depending on the assay to be performed later on, the pregel was extruded in 40 mm Petri dishes or in a 24-multiwell plate and left to incubate for 1 h at 37 °C in a humid atmosphere.

#### 4.2.3. Crosslinking of L-dECM Hydrogels with Genipin

Following the protocol described in [23], 5 mM, 10 mM, and 20 mM genipin (Challenge Bioproducts, Douliu, Taiwan) solutions were prepared. The mixture was kept at 40 °C under moderate stirring until complete dissolution of genipin was observed. The solutions were filtered to remove any precipitate with a syringe and a 0.22 µm pore size filter and left to rest for 16 h at RT. The corresponding genipin solution was then added to the hydrogel after gelation and left for 72 h at RT until complete crosslinking, indicated by a change in color from white to dark blue. The hydrogels were then washed with 1X PBS 3 times to remove the excess genipin.

### 4.3. Protocol for Obtaining Hydrogel Cryosections

#### 4.3.1. Method for Infiltration of Hydrogels for Cryosectioning

One of the critical steps in hydrogel preparation is their freezing. Direct freezing is not possible due to the internal formation of crystals, which damage the structure and make it difficult to obtain clean cuts [37]. Therefore, a hydrogel infiltration method described in [38], with minor modifications, was applied. This method has been previously used for fixed hydrogels; however, application to unfixed and crosslinked hydrogels has not been explored. For this protocol, we used 500 µL/well L-dECM genipin crosslinked hydrogels generated in a 24-well plate as a template. The thickness of each resulting hydrogel was calculated by volume/surface area, resulting in an expected height of 2631 µm. After gelation, 1 mL/well of genipin solution was added, with the cross-linking time depending on the experiment. The first day of the protocol involved infiltrating sucrose (Sigma-Aldrich, St. Louis, MO, USA) into the hydrogel, thus acting as a cryoprotectant. In brief, 1 mL of 5% sucrose (g/mL) was added to each well for 1 h at RT. The solution was removed and replaced by adding 0.5 mL 20% sucrose (g/mL) every 30 min up to 3 times. It was replaced again to add 1 mL of fresh 20% sucrose and incubated for 16 h at 4 °C. On the second day of the protocol, the previous solution was removed and 1.5 mL/well of sucrose-OCT (ThermoFisher Scientific, Waltham, MA, USA) solution (2:1 concentration) was added and incubated for 1 h at RT. The solution was then removed, and 0.5 mL of OCT was added and incubated again for 1 h. OCT was replaced with 1 mL of fresh volume and incubated 24 h at 4 °C. Finally, on the third day, the incubated solution was replaced again with 1 mL of OCT, incubated 3 h at RT and then frozen.

#### 4.3.2. Mounting and Freezing of OCT Infiltrated Hydrogels

Cryomolds (Sakura Finetek, The Netherlands) were prepared with a thin layer of OCT and placed in the cryostat (Leica Biosystems, Nussloch, Germany, model: CM3050S) at a chamber temperature (CT) of −23 °C. In turn, the hydrogels were removed from the 24-plate with a spatula and deposited on the cover of the multiwell plate, allowing them to freeze at the same temperature. Once the OCT was frozen, the hydrogel was easily peeled off with a spatula, placed in the middle of the cryomold, and completely covered with OCT. Care was taken to avoid bubbles, or remove them with a fine-gauge needle. The hydrogels were left in the cryostat chamber for at least 2 h, and the color of the OCT was confirmed to be uniformly white indicating complete freezing. Gels were then stored at −80 °C until use. For cutting, cryomolds were removed from −80 °C and placed in the cryostat (T = −23 °C) for at least 30 min prior to mounting, to be cut as previously recommended [39]. Hydrogel slides of 100 µm thickness were sectioned and deposited on a positively charged glass slide (SuperFrost Plus, ThermoFisher Scientific, Waltham, MA, USA). The samples were cut at an object temperature (OT) of −20 °C and CT of −23 °C.

#### 4.3.3. Rehydration of the Slides

Within the cutting steps, the most critical one involves removal of OCT at RT followed by rehydration of the slide, so that it does not collapse and maintains the same mechanical characteristics as the unfrozen hydrogel. For this reason, time-dependent hydration curves of the slides were performed. After cutting the sample, we waited different times (5, 10, 20, 30, 40, and 60 min) and then rehydrated the slides with 1X PBS. To completely remove the OCT, 3 washes of 5 min with 1X PBS were performed. Between each wash, the slide is decanted to passively remove the solution without damaging the sample. The samples were measured by AFM to obtain the hydrogel mechanical properties at each rehydration time and the equation obtained was used to determine the optimal rehydration time to achieve the same stiffness as for unfrozen hydrogels.

### 4.4. Sterilization of Hydrogels

After a bibliographic search on the most used sample sterilization approaches, hydrogel sterilization with 70% (*v*/*v*) ethanol was chosen. Hydrogels were exposed to 70% ethanol for either 20 min or 24 h. After sterilization, they were washed with distilled water for 5 min 3 times to ensure complete removal of ethanol.

### 4.5. Mechanical Measurements by AFM

Measurements were performed with a custom AFM mounted on an inverted optical microscope where the cantilevers had nominal spring (k) values of 0.01 N/m and a 4.5 µm diameter silicon nitride bead attached to its end (Novascan Technologies, Ames, IA, USA). Measurement of vertical displacement (z) as well as 3D cantilever displacement was achieved by coupling piezo actuators to strain gauge sensors (Physik Instrumente, Germany). The cantilever deflection (d) was evaluated by means of a quadrant photodiode (S4349, Hamamatsu, Japan). All measurements were performed with the sample immersed in 1X PBS and at RT. Before starting the measurements on the sample, the slope of the deflection-displacement (d-z) curve was obtained from the indentation on a bare area considered to be of infinite stiffness. This curve was used to calibrate the relationship between the photodiode signal and the cantilever deflection. For measurements on the sample, the tip was macroscopically placed over the sample, and d-z curves were recorded at a constant speed of 20 µm/s and a frequency of 1 Hz. A minimum of 4 zones at least 50–100 µm apart were recorded. In each zone, 5 points at least 20 µm apart were recorded for a total of 15–20 points per sample.

### 4.6. AFM Data Processing

To calculate E from the d-z curves, the following data analysis steps were carried out. First, the deflection of the cantilever was converted to force (*F*) using Hooke’s law, defined by the following equation [40]:

(1) $$F=k*\Delta_{zt}$$

where *k* is the spring constant and Δ*_zt_* is the deflection of the cantilever, produced by the local resistance of the sample to deformation during contact with the tip. On the other hand, the local sample deformation or indentation (*δ*) is calculated as follows [40]:

(2) δ=z−d

where *z* is the vertical displacement beyond the tip-sample contact point. Finally, a suitable elastic contact model that takes into account the actual tip geometry is fitted to the data (*F*, *δ*). Since the typology of our cantilever is spherical and in line with previous published work [41], we use the Hertz model, which describes the interaction between a sphere and a semi-infinite space (sample). In that case, Young’s modulus is described by the following equation [40]:

(3) $$F=\frac{4}{3\left(1-{ⱴ}^{2}\right)}\;E_{m}\;R^{\frac{1}{2}}\;\delta^{\frac{3}{2}}$$

where *R* is the radius of the sphere, ⱴ is the Poisson’s ratio (assumed to be 0.5), and *E_m_* the microscale stiffness of the sample. For each d-z curve obtained, the contact point was first identified [42] and *E_m_* was calculated by non-linear least squares fitting using custom developed scripts in Matlab R2023a.

### 4.7. Viability/Cytotoxicity Test and F-Actin Staining

Cultured cells were subjected to Live/Dead staining (consisting of calcein/ethidium; ThermoScientific, Waltham, MA, USA) to check their viability after 72 h in culture on the cryosection dECM hydrogels. Cells were fixed with 4% PFA (Sigma-Aldrich, St. Louis, MO, USA) for 15 min, and washed with 1X PBS 3 times.

For F-actin fibroblast were fixed in 4% PFA (Sigma-Aldrich, St. Louis, MO, USA) for 15 min and permeabilized with 0.1% triton X-100 (Sigma-Aldrich, St. Louis, MO, USA) for 20 min. Subsequently, the samples were incubated with Phalloidin-iFluor 488 Reagent (ab176753, Abcam, Cambridge, UK) at a dilution of 1:750 for 45 min at room temperature. The samples were then washed 3 times with PBS1x and incubated with a 1:1000 solution of DAPI (NucBlue, ThermoScientific, Waltham, MA, USA). The samples were then washed with PBS 3 times and transferred with the seeded surfaces facing down onto a coverslip using Fluoromount-G mounting medium (ThermoScientific, Waltham, MA, USA).

For fluorescence imaging acquisition, a Nikon D-Eclipse Ci confocal microscope was used in conjunction with a ×10 Plan Fluor (Nikon) for Live/Dead staining-(epifluorescence mode) and ×20 Plan Apo immersion oil objective (Nikon) for F-actin staining (z-stack confocal mode).

### 4.8. Statistical Analysis

Statistical analysis and the generation of graphs was performed using GraphPad Prism (GraphPad Prism Software version 8.0.2, Boston, MA, USA). All data are plotted as median ± interquartile range, unless otherwise specified. The normal and lognormal distribution of the data was tested using Shapiro–Wilk tests for normality and log-normality, and log-normality of the data was confirmed. For testing between two groups, unpaired parametric Student’s *t*-tests were used for all comparisons. For testing between more than two groups, a one-way analysis of variance (ANOVA) with Tukey post-doc multiple comparisons test was used. Differences were considered statistically significant for *p*-values < 0.05.

## Figures and Tables

**Table 1:** Sources of variability of the slides. The results are expressed as percentage covariance, for inter-slide variability it is represented as mean covariance ± STD. Inter-gel variability is expressed as the mean of the covariances of both hydrogels.

Variability Sources		
Intergel	Variability due to macroscopic gel preparation another factors (relative humidity, temperature, systematic errors, etc.)	6.3%
Intrarramp	Quality of single force-indentation curves acquired by AFM.	8.2% (4.5–14.4)
Interslide	Variability due to hydrogel cutting cryoslide protocol (freezing, cutting and rehydration).	17.5 ± 9.5%
Intraslide	Variability due to regional heterogeneity of source sample (genipin crosslinked L-dECM hydrogel).	52.6% (34.9–72.2)

## Data Availability

Data are available upon reasonable request. Our AFM setup is custom-built and as such it outputs the data in a non-standard way.
